# Supplementary material for: Polyketide synthase gene clusters in the ericoid mycorrhizal fungus Oidiodendron maius: genome-wide prediction, expression profiling, and functional characterization of knockout mutants
Source: Front Plant Sci. 2026 Sep 16;17:1924430. doi: 10.3389/fpls.2026.1924430 (PMC13623903; doi:10.3389/fpls.2026.1924430)
Supplement: Supplementary file 1 [file SupplementaryFile1.zip › Supplementary Materials/Supplementary Folder 1.zip!/Oidma1_AssemblyScaffolds_Repeatmasked/knownclusterblast/cluster24/jgi.p_Oidma1_42801_mibig_hits.html]

| MIBiG Protein | Description | MIBiG Cluster | MiBiG Product | % ID | % Coverage | BLAST Score | E-value |
| --- | --- | --- | --- | --- | --- | --- | --- |
| AFY23020.1 | betaine\_aldehyde\_dehydrogenase | BGC0000274 | Tetarimycin\_biosynthetic\_gene\_cluster | 32 | 97.7687626775 | 237 | 4.6e-62 |
| ABW71833.1 | putative\_aldehyde\_dehydrogease | BGC0000303 | Anthramycin\_biosynthetic\_gene\_cluster | 34 | 97.1602434077 | 225 | 1.4e-58 |
| AFT91402.1 | hypothetical\_protein | BGC0000338\_c2 | Echinocandin\_B\_biosynthetic\_gene\_cluster | 30 | 98.1744421907 | 225 | 1.8e-58 |
| CAQ52616.1 | benzaldehyde\_dehydrogenase | BGC0001066 | Kendomycin\_biosynthetic\_gene\_cluster | 33 | 92.9006085193 | 220 | 3.4e-57 |
| ABB90277.1 | aldehyde\_dehydrogenase | BGC0001057 | Zearalenone\_biosynthetic\_gene\_cluster | 30 | 95.1318458418 | 219 | 9.9e-57 |
| BAK64640.1 | putative\_aldehyde\_dehydrogenase | BGC0000135 | Reveromycin\_biosynthetic\_gene\_cluster | 33 | 88.8438133874 | 196 | 8.9e-50 |
| ABC35115.1 | aldehyde\_dehydrogenase\_family\_protein | BGC0001102 | Malleilactone\_biosynthetic\_gene\_cluster | 30 | 96.1460446247 | 193 | 4.4e-49 |
| AAS47555.1 | putative\_aldehyde\_dehydrogenase | BGC0001108 | Pederin\_biosynthetic\_gene\_cluster | 29 | 94.7261663286 | 183 | 4.6e-46 |
| ALI92651.1 | CitD\_dehydrogenase | BGC0001338 | Citrinin\_biosynthetic\_gene\_cluster | 29 | 91.2778904665 | 177 | 3.3e-44 |
| ABY65994.1 | aldehyde\_dehydrogenase | BGC0001008 | Maduropeptin\_biosynthetic\_gene\_cluster | 29 | 94.3204868154 | 172 | 1.1e-42 |
| AAD32735.1 | MmcL | BGC0000915 | Mitomycin\_biosynthetic\_gene\_cluster | 29 | 91.2778904665 | 168 | 2e-41 |
